# Long noncoding RNA 01534 maintains cancer stemness by downregulating endoplasmic reticulum stress response in colorectal cancer

**DOI:** 10.1002/ags3.12649

**Published:** 2022-12-29

**Authors:** Momoko Ichihara, Hidekazu Takahashi, Naohiro Nishida, Cristina Ivan, Daisuke Okuzaki, Yuhki Yokoyama, Masahisa Ohtsuka, Norikatsu Miyoshi, Mamoru Uemura, Shinji Tanaka, George Adrian Calin, Masaki Mori, Yuichiro Doki, Hidetoshi Eguchi, Hirofumi Yamamoto

**Affiliations:** ^1^ Department of Surgery, Gastroenterological Surgery, Graduate School of Medicine Osaka University Suita Osaka Japan; ^2^ Department of Medical Oncology Osaka International Cancer Institute Osaka Osaka Japan; ^3^ Department of Experimental Therapeutics The University of Texas, MD Anderson Cancer Center Houston Texas USA; ^4^ Genome Information Research Centre Research Institute for Microbial Diseases, Osaka University Suita Osaka Japan; ^5^ Department of Molecular Pathology, Division of Health Sciences, Graduate School of Medicine Osaka University Suita Osaka Japan; ^6^ Department of Surgery Kindai University Nara Hospital Ikoma Nara Japan; ^7^ Departments of Molecular Oncology, Graduate School of Medicine Tokyo Medical and Dental University Tokyo Japan; ^8^ Tokai University, Graduate School of Medicine Isehara Kanagawa Japan

**Keywords:** cancer stemness, colorectal cancer, endoplasmic reticulum stress, LINC01534, long noncoding RNA

## Abstract

**Background:**

Studies have shown that cancer stemness and the endoplasmic reticulum (ER) stress response are inversely regulated in colorectal cancer (CRC), but the mechanism has not been fully clarified. Long noncoding RNAs (lncRNAs) play key roles in cancer progression and metastasis. In this study we investigated lncRNA 01534 (LINC01534) as a possible modulator between cancer stemness and ER stress response.

**Methods:**

In vitro experiments using CRC cell lines were performed to explore a possible role of LINC01534. The expression of LINC01534 in clinical CRC samples was assessed by quantitative reverse transcription‐polymerase chain reaction (qRT‐PCR) and in situ hybridization.

**Results:**

Silencing LINC01534 led to suppression of cell proliferation, invasiveness, and cell cycle progression at the G2‐M phase, and promoted apoptosis. Moreover, we found that silencing LINC01534 suppressed cancer stemness, while it activated the ER stress response, especially through the PERK/eIF2α signaling pathway. In situ hybridization revealed LINC01534 was expressed in tumor cells and upregulated in CRC tissues compared with normal epithelium. A survival survey indicated that high LINC01534 expression was significantly associated with shorter overall survival in 187 CRC patients.

**Conclusion:**

This is the first report on LINC01534 in human cancer. Our findings suggest that LINC01534 may be an important modulator of the maintenance of cancer stemness and suppression of the ER stress response, and that it could be a novel prognostic factor in CRC.

## Graphical abstract

Long noncoding RNA 01534 (LINC01534) had a function in maintenance of cancer stemness and coordinated suppression of endoplasmic reticulum stress response. Moreover, we found that high expression of LINC01534 was associated with poor prognosis of colorectal cancer (CRC) patients. This study suggested that targeting LINC01534 is a potential therapeutic option against CRC that may improve outcome in CRC patients.

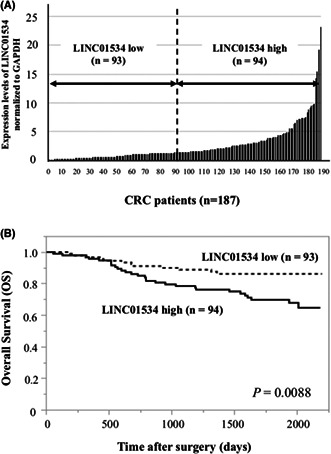

## 1 INTRODUCTION

The incidence of colorectal cancer (CRC) has increased over recent years, and CRC is one of the most common gastrointestinal cancers worldwide, with the third highest morbidity (10.2%) and the second highest mortality (9.2%) in 2018.^1^ Despite advances in therapeutic strategies—including surgical technology, radiotherapy, chemotherapy, and molecular‐targeted therapy—the 5‐y survival rate remains <65%; thus, the molecular basis of CRC must be further investigated.^2^, ^3^

Protein coding genes account for <2% of human genes, and the nonprotein‐coding portion of the human genome was long considered “junk DNA.”^4^, ^5^ However, noncoding RNAs (ncRNAs) are now the focus of increasing attention.^5^ Long noncoding RNAs (lncRNAs) are defined as transcripts of over 200 nucleotides, in contrast to the small ncRNA group, which includes microRNAs (miRNAs).^6^ LncRNAs have recently been found to play key roles in biological processes and pathological conditions related to cancer development.^7^, ^8^ LncRNAs regulate gene expression through many mechanisms, including acting as scaffolds for chromatin modifiers, microRNA sponges, transcriptional regulators, protein decoys, and enhancers.^4^, ^6^ Several lncRNAs, including HOTAIR and LUCRC, have been reported as prognostic factors in CRC.^4^, ^8^ These lncRNAs control tumor cell growth, migration, and invasion by affecting cell cycle progression and the epithelial–mesenchymal transition through altered signaling in multiple pathways including those of p53, AKT/mTOR, JAK/STAT, and NF‐kB.^4^, ^6^, ^9^ However, the functions of most lncRNAs remain unclear.^7^

A recent report demonstrated high expression of lncRNA 01534 (LINC01534) in the inflammatory disease osteoarthritis, and showed that LINC01534 promotes an inflammatory response through proinflammatory factors, such as TNF‐α and IL‐6.^10^ To date, however, we are not aware of any reports that describe LINC01534 in human cancer. We previously demonstrated that low proteasome activity cells (LPACs) that were visualized by ZsGreen fused to the carboxyl terminal degron of ornithine decarboxylase (ODC) can serve as a cancer stem cell (CSC) model in colon cancer cells.^11^ Using ODC degron‐transduced HCT116 cells as LPACs, we found by RNA sequencing that LINC01534 was one of the upregulated lncRNAs. Notably, RNA sequence analysis revealed that siRNA treatment for LINC01534 restored the endoplasmic reticulum (ER) stress response in CRC cells. This particularly drew our attention because several studies have reported that stemness and the ER stress response are inversely regulated in colonic epithelium and CRC cells.^12^, ^13^ The ER stress response is an adaptation to many stresses—including nutrient and lipid deprivation, hypoxia, and acidic extracellular pH—and its role in cancer remains largely unknown.^14^, ^15^, ^16^

In an effort to reveal a role of LINC01534 in CRC, we performed in vitro experiments including cancer stemness and the ER stress response and assessed its clinical significance in the survival of 187 CRC patients. Overall, the present study reveals many important aspects of LINC01534 in CRC in terms of its expression, function, and clinical relevance.

## 2 METHODS

### 2.1 Cell lines and culture conditions

The human CRC cell lines HCT116 and RKO were purchased from the American Type Culture Collection. Cells were cultured in Dulbecco's modified Eagle's medium (DMEM) supplemented with 10% fetal bovine serum (FBS) and 100 U/ml penicillin/streptomycin. For glucose‐ or glutamine‐deprived cultures, DMEM/no glucose (Thermo Fisher Scientific) or DMEM/low glucose/pyruvate/no glutamine/no phenol red (Thermo Fisher Scientific) were used. Cultures were maintained at 37°C in a humid incubator with 5% CO_2_.

### 2.2 Retroviral transduction of the degron reporter

The degron sequence of ornithine decarboxylase (ODC) is recognized directly by proteasomes, leading to the destruction of the involved protein.^11^, ^17^ The retroviral expression vector pQCXIN‐ZsGreen‐cODC, containing the green fluorescent ZsGreen‐labeled ODC degron (Gdeg), was kindly provided by Dr Frank Pajonk (Jonsson Comprehensive Cancer Center, UCLA). LPACs express ZsGreen and could be detected by fluorescent microscopy or flow cytometry.

The plasmid was transfected into platinum retroviral packaging cells using Lipofectamine 2000 (Thermo Fisher Scientific). The retrovirus collected from the supernatant was used for infection. Stable transfectants were selected with G418 solution (Sigma‐Aldrich) and maintained in 0.1 mg/ml G418 solution.

### 2.3 Small interfering RNA and transfection

We purchased siLINC01534 from Thermo Fisher Scientific, and negative control siRNA from Gene Design (Osaka, Japan). The target sequences were as follows:

siLINC01534‐1: sense, 5′‐AUCGAGUGGUGGAAUAAAA[dT][dT]‐3′; antisense, 5′‐UUUUAUUCCACCACUCGAU[dT][dA]‐3′.

siLINC01534‐2: sense, 5′‐GAACGAGGCUUUUCGAGUU[dT][dT]‐3′; antisense, 5′‐AACUCGAAAAGCCUCGUUC[dC][dG]‐3′.

si negative control (NC): sense, AUCCGCGCGAUAGUACGUA, antisense, UACGUACUAUCGCGCGGAU.

Transfection was performed using Lipofectamine RNAiMax (Thermo Fisher Scientific).

### 2.4 Cell proliferation assay

Cells were seeded at a density of 3000–5000/well in 96‐well plates, and cultured for 24–72 h. The viable cell number was counted using Cell Counting Kit‐8 (Dojindo Laboratories).

### 2.5 Cell cycle assay

Cells were starved for 48 h (HCT116 cells) or 72 h (RKO cells). Next, cells were transfected with negative control siRNA or siLINC01534. Cells were restimulated by changing the medium with 10% FBS after 24 h (HCT116 cells) or 6 h (RKO), and collected at the indicated timepoints. Cells were washed twice with phosphate‐buffered saline (PBS) and fixed in 70% ethanol at 4°C for 6 h. The fixed cells were incubated with RNase (Sigma‐Aldrich) for 10 min at 37°C, treated with propidium iodide (Sigma‐Aldrich) for 20 min at room temperature in the dark, and analyzed by flow cytometry using a BD FACS Canto Clinical Flow Cytometry System (BD Biosciences, Franklin Lakes, NJ, USA). The percentages of cells in different phases of the cell cycle were determined using the FlowJo computer program version 10.5.3 (BD Biosciences).

### 2.6 Invasion assay

Invasion assays were performed using polyethylene terephthalate membranes (8‐μm pore size) in a BioCoat Matrigel Invasion Chamber (Corning Inc.), as previously described.^18^ Briefly, a 0.5‐ml cell suspension (DMEM including 2 × 10^5^ cells/ml for HCT116 cells and 3 × 10^5^ cells/ml for RKO) was added to the upper well chamber. After removal of noninvading cells from the upper side of the membranes, the invading cells on the lower side were fixed and stained with Diff‐Quik (Sysmex) for counting.

### 2.7 Apoptosis assay

After 12 h of transfection with negative control siRNA or siLINC01534, apoptosis of CRC cells was measured using an Annexin V‐FITC Apoptosis Kit (BioVision), following the manufacturer's protocol. Cells were subsequently analyzed by flow cytometry using a BD FACS Canto Clinical Flow Cytometry System (BD Biosciences).

### 2.8 Quantitative reverse transcription‐polymerase chain reaction (qRT‐PCR)

TRIzol RNA Isolation Reagent (Thermo Fisher Scientific) was used to extract total RNA from the cells.^19^ cDNA was synthesized from 10 ng total RNA using the Rever Tra Ace qRT Master Mix (Toyobo Life Science). Quantitative PCR was performed in a Light Cycle 2.0 System (Roche Applied Science). The amplification conditions were as follows: initial denaturation at 95°C for 10 min, followed by 45 cycles of denaturation at 95°C for 10 s, annealing at 60°C for 10 s, and extension at 72°C for 10 sec, as previously described.^20^ Data were normalized to the expression of glyceraldehyde‐3‐phosphate dehydrogenase (GAPDH). The sequences of the primers are listed in Table S1.

### 2.9 Sphere formation assay

HCT116 cells were seeded in 96‐well ultralow attachment plates (Corning) at a density of 1000 cells/well. These cells were transfected with negative control siRNA or siLINC01534, and cultured in DMEM/F‐12 serum‐free medium supplemented with 20 ng/ml epithelial growth factor and 10 ng/ml fibroblast growth factor‐2 at 37°C. At 6 d after seeding, the number of spheres of diameter ≥150 μm was counted.

### 2.10 Chemosensitivity assay

HCT116 cells were seeded at a density of 4000 cells/well in 96‐well plates, cultured for 24 h, and then transfected with negative control siRNA or siLINC01534. After 24 h of transfection, cells were treated with 5‐fluorouracil (5‐FU) (Nacalai Tesque Inc.) for 48 or 96 h. The viable cell number was counted with a Cell Counting Kit‐8.

### 2.11 RNA sequencing

Total RNA was extracted from cells using the miRNeasy Mini Kit (Qiagen). The library was prepared with a TruSeq Stranded mRNA Sample Prep Kit (Illumina). The RNA samples were subjected to whole‐transcriptome sequencing using the Illumina HiSeq 2500 platform in 75‐base single‐end mode. Base calling was performed using Illumina Casava 1.8.2 software, and the sequenced reads were mapped to human reference genome sequences (hg19) using TopHat version 2.0.13, combined with Bowtie2 version 2.2.3 and SAMtools version 0.1.19. The fragments per kilobase of exon per million mapped fragments (FPKMs) were calculated using Cuffnorm version 2.2.1. By comparison with triplicate cultures, we identified a series of genes that were enhanced (>2.0‐fold) or reduced (<2.0‐fold) for further gene expression analysis. The raw data from this study were submitted under Gene Expression Omnibus (GEO) accession number GSE179624 (https://www.ncbi.nlm.nih.gov/geo/query/acc.cgi?acc=GSE179624, enter ilktygmgdpglxex into the box). GSEA version 3.0 was used to identify gene sets that were significantly altered by the addition of siLINC01534 from the Gene Ontology database. Gene sets were considered activated if the false discovery rate (FDR) q‐value was less than 0.05.

### 2.12 Western blot analysis

Whole cells were lysed in RIPA buffer (Thermo Fisher Scientific) including a phosphatase and protease inhibitor cocktail (Thermo Fisher Scientific). Lysates were separated by electrophoresis, incubated with primary antibody overnight at 4°C, and then incubated for 1 h at room temperature with antirabbit immunoglobulin (Ig) G horseradish peroxidase‐linked antibody or with antimouse IgG horseradish peroxidase‐linked antibody. They were then transferred to polyvinylidene difluoride (PVDF) membranes. The antigen–antibody complex was detected using Amersham Enhanced Chemiluminescence Prime Western Blotting Detection Reagent (GE Healthcare Biosciences) with an Image Quant LAS4000 (Fujifilm). The primary antibodies are listed in Table S2.

### 2.13 RNAscope in situ hybridization (ISH) assay

RNAscope ISH assays were performed using an RNAscope 2.0 High‐definition Assay Kit (Advanced Cell Diagnostics). In tissue sections, the LINC01534 expression levels were scored based on the number of dots or clusters derived from RNA molecules in the cells, as follows; Score 0: 0 dot/cell; score 1: 1–3 dots/cell; score 2: 4–9 dots/cell, occasional cluster formation present; and score 3: ≥10 dots/cell, frequent cluster formation present.

### 2.14 Clinical samples

A total of 187 CRC clinical samples were collected from patients who underwent surgery between June 2003 and September 2005 at Osaka University Hospital and its related hospitals. Seven patients had received neoadjuvant chemotherapy and/or radiation due to distant metastases. All specimens were immediately frozen and stored at −80°C until RNA extraction. LINC01534 expression in the CRC tissue sample was measured by qRT‐PCR and GAPDH RNA expression was used for normalization. The Union for International Cancer Control classification was used for patient staging. The mean follow‐up time of patients was 59.7 mo for overall survival (OS). Written informed consent was obtained from all patients, and the study protocol was in accordance with the Declaration of Helsinki, the Japanese Ethical Guidelines for Human Genome/Gene Analysis Research, and the Ethical Guidelines for Medical and Health Research Involving Human Subjects in Osaka University (Permission No. #15144).

We also obtained gene expression and clinical data from The Cancer Genome Atlas Project (TCGA; http://tcga‐data.nci.nih.gov/) for colon cancer patients containing a total of 367 clinical samples.

### 2.15 Statistical analysis

Statistical analyses were performed using Student's *t*‐test, the chi‐square test, or Fisher's exact test for categorical data, and the Mann–Whitney *U*‐test for nonparametric data. Correlations were assessed by calculating the Pearson's correlation coefficient. *p* < 0.05 was considered statistically significant. Patient survival was examined with the Kaplan–Meier method, and the log‐rank test was used to determine significance. Data are shown as the mean ± SD of the indicated number of experiments. Statistical analyses were performed using JMP Pro 15.0 (SAS Institute Inc.).

## 3 RESULTS

### 3.1 LINC01534 expression was upregulated in LPACs

Our comprehensive transcriptome analysis by lncRNA sequencing comparing LPACs and non‐LPACs in HCT116 cells identified 34 lncRNAs that were upregulated (>2.0‐fold) in LPACs (Figure S1A). The TCGA dataset indicated that high expression of LINC01534 (*n* = 235) was significantly associated with shorter OS when compared with low LINC01534 expression (*n* = 132) (*p =* 0.0213; Figure S1B).

### 3.2 Silencing of LINC01534 inhibited cell proliferation

**FIGURE 1:**
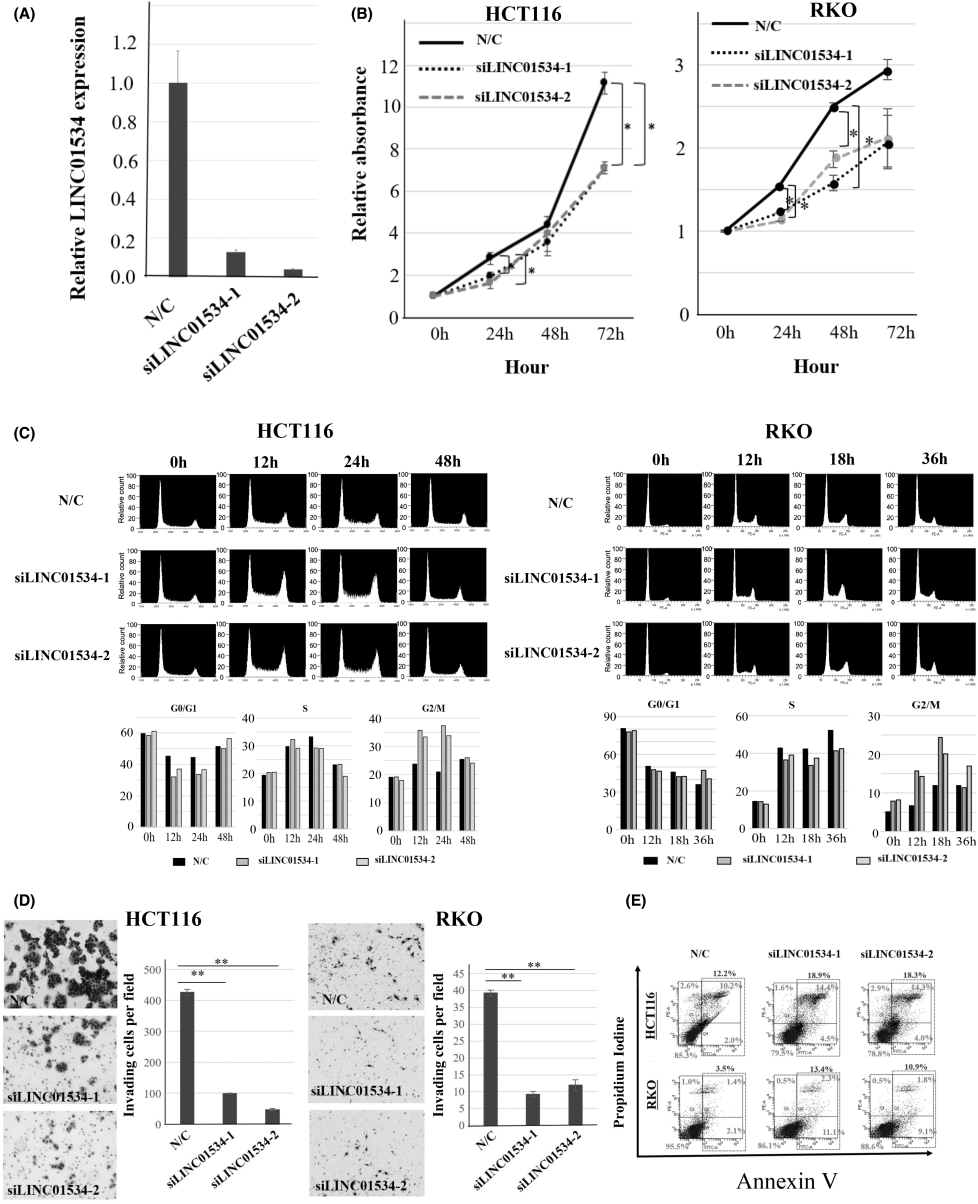
Effects of LINC01534 silencing on the proliferation, cell cycle progression, invasiveness, and apoptosis of colon cancer cells. (A) LINC01534 expression in the HCT116 cells transfected with negative control siRNA, siLINC01534‐1, and siLINC01534‐2. (B) Proliferation assay showing that siRNAs that silence LINC01534 significantly reduced proliferation compared with the negative control siRNA. *p < 0.05. Data presented as mean ± SD. (C) Cell cycle analysis by flow cytometry after resupplementation with FBS in control cells and LINC01534‐silenced cells. SiLINC01534 treatment induced G2‐M arrest in HCT116 and RKO cells. Relative count is shown in the y‐axis; the maximum value of y‐axis was considered 100. (D) Invasion assay. Cells passing through the upper chamber were stained with Diff‐Quik. SiLINC01534 treatment significantly inhibited the invasiveness of HCT116 and RKO cells. **p < 0.001. Data presented as mean ± SD. (E) Apoptosis assays. HCT116 (upper) and RKO (lower) cell lines were transfected with the indicated siRNAs. SiLINC01534 treatment increased apoptosis. The percentage of early apoptotic cells is shown in the lower‐right field and the percentage of late apoptotic cells is shown in the upper‐right field. The total percentage of apoptotic cells is shown on the top

QRT‐PCR indicated that the CRC cell lines HCT116 and RKO expressed relatively high levels of LINC01534 (Figure S2). We confirmed that siLINC01534‐1 and siLINC01534‐2 reduced LINC01534 expression by >85% compared with the level in cells treated with the negative control siRNA (Figure 1A). Compared with negative control siRNA treatment, silencing of LINC01534 significantly suppressed proliferation of the HCT116 and RKO cell lines (*p <* 0.05, Figure 1B).

### 3.3 Silencing of LINC01534 causes cell cycle arrest at the G2‐M transition

Compared with the negative control group, LINC01534‐silenced HCT116 cells exhibited a higher percentage of cells in G2‐M phase at 12 and 24 h after restimulation with 10% FBS. LINC01534‐silenced RKO cells also exhibited a higher percentage in G2‐M phase at 12 and 18 h (Figure 1C). Western blot analysis revealed that silencing LINC01534 led to decreased expressions of several G2‐M accelerators, including cdc25B, cdc25C, and cdc2 in HCT116 cells (Figure S3).

### 3.4 Silencing of LINC01534 inhibits invasive capability and induces apoptosis

Silencing LINC01534 markedly reduced the invasiveness of HCT116 and RKO cells (*p <* 0.001; Figure 1D). The annexin V assay showed an increase in apoptosis in both cell lines (Figure 1E).

### 3.5 Silencing LINC01534 suppresses cancer stemness

**FIGURE 2:**
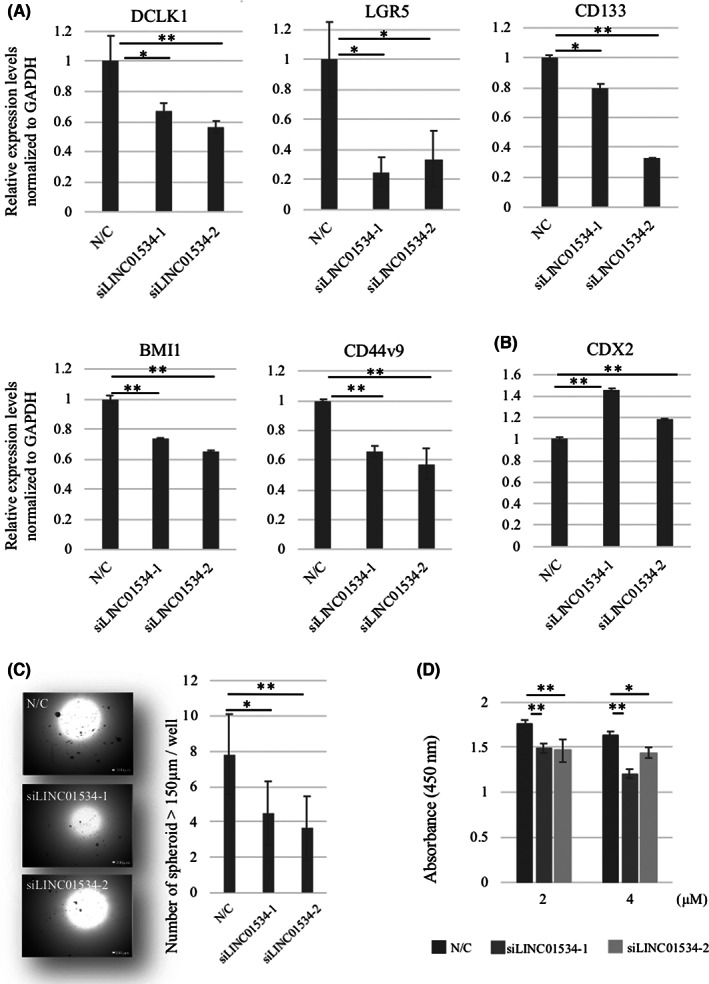
SiLINC01534 treatment suppressed cancer stemness in HCT116 cells. (A) qRT‐PCR showed that transfection with siLINC01534 significantly suppressed the expression of stem cell markers including DCLK1, LGR5, CD133, BMI1, and CD44v9. *p < 0.05. **p < 0.001. Data presented as mean ± SD. (B) qRT‐PCR showed that transfection with siLINC01534 significantly increased the expression of the epithelial differentiation marker, CDX2. **p < 0.001. Data presented as mean ± SD. (C) Sphere formation assays revealed that siLIN01534 transfection significantly decreased the number of spheres (≥150 μm) on d 6. *p < 0.05. **p < 0.001. Data presented as mean ± SD. Scale bar, 100 μm. (D) Sensitivities to 5‐FU in the cells transfected with negative controls siRNA and siLINC01534. Cell viability was measured by CCK‐8 assay after 48 h with 5‐FU treatment. The LINC01534‐silenced cells had higher sensitivity to 5‐FU than the negative control cells. *p < 0.05. **p < 0.001. Data presented as mean ± SD

We then examined cancer stemness by silencing LINC01534 in HCT116 cells, which reportedly retain cancer stem‐cell properties.^21^, ^22^, ^23^ Silencing LINC01534 significantly suppressed stem cell markers, including DCLK1, LGR5, CD133, BMI, and CD44v9, and increased the epithelial differentiation marker, CDX2 (*p < 0.05, **p < 0.001, Figure 2A,B). Sphere formation assays showed that the number of spheroids was significantly decreased by silencing LINC01534 (*p < 0.05, **p < 0.001, Figure 2C). We then examined the effects of silencing LINC01534 on the sensitivity of chemotherapy. After 48 h of treatment with 5‐FU at concentrations of 2 and 4 μM (around the IC50^24^, ^25^), LINC01534‐silenced HCT116 cells had significantly higher sensitivity to 5‐FU than did the control cells (*p < 0.05, **p < 0.001, Figure 2D). When the cells were treated with 5‐FU at lower concentrations (0.25, 0.5, 1.0 μM) for 96 h, siLINC01534 treatment led to increased chemosensitivity at each concentration (*p < 0.05. **p < 0.001, Figure S4).

### 3.6 Silencing of LINC01534 activates the endoplasmic reticulum (ER) stress response

**FIGURE 3:**
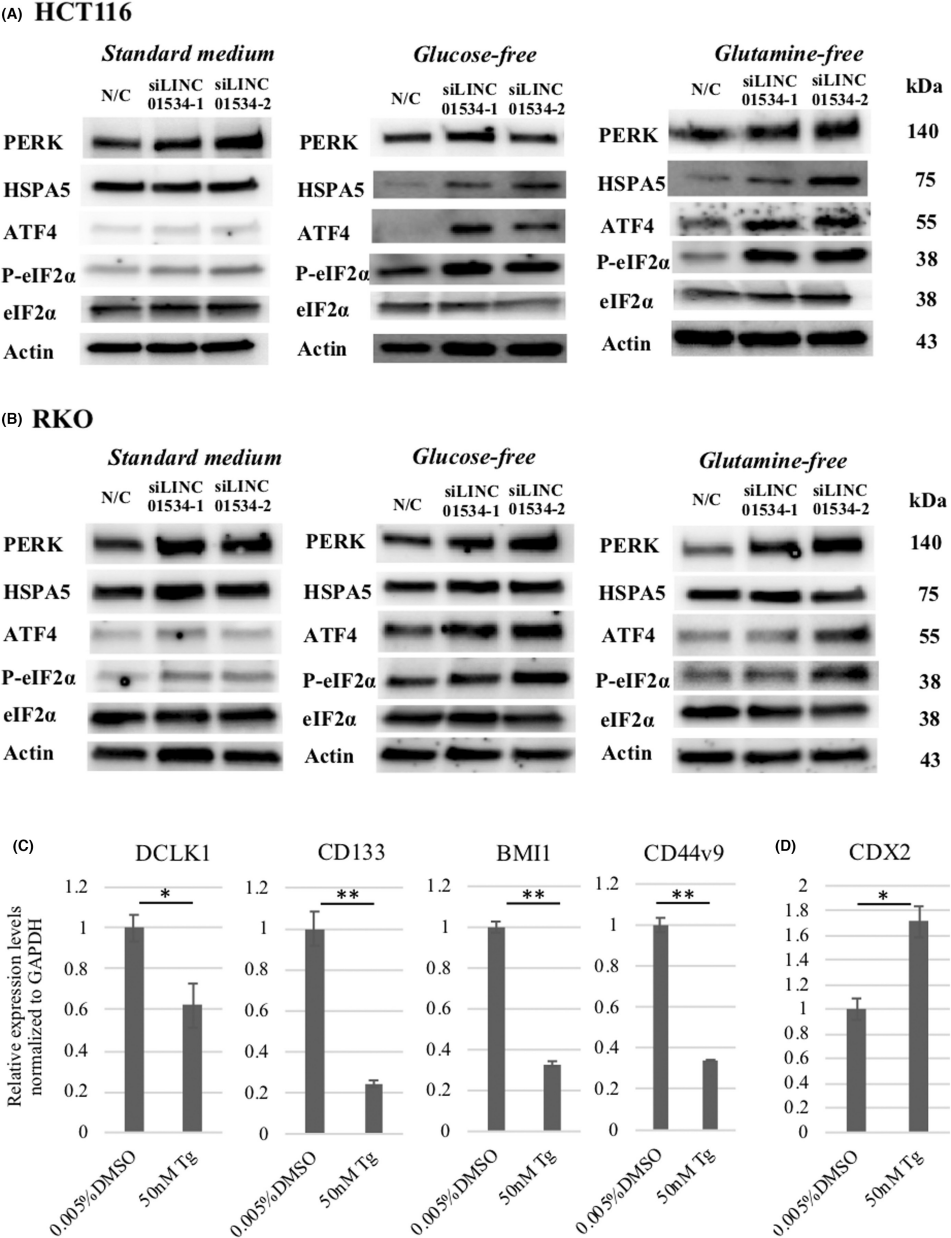
SiLINC01534 treatment activated the unfolded protein response in the PERK/p‐eIF2α signaling pathway under standard or glucose‐ or glutamine‐deprived conditions. (A) Expression of the proteins PERK, HSPA5, ATF4, P‐eIF2α, eIF2α, and actin was examined by western blot analysis 9 h after transfection with negative controls siRNA, siLINC01534‐1, or siLINC01534‐2, in HCT116 cells. (B) Expression of the proteins PERK, HSPA5, ATF4, P‐eIF2α, eIF2α, and actin was examined by western blot analysis 24 h after transfection with the negative controls, siLINC01534‐1, or siLINC01534‐2 in RKO cells. Western blot analysis showed that silencing LINC01534 upregulated molecules involved in the PERK pathway, represented by phosphorylated eIF2α. In glucose‐ or glutamine‐deprived conditions the activation was more evident. The β‐actin bands served as a loading control. (C) qRT‐PCR showed that treatment with a UPR inducer, thapsigargin (Tg), significantly suppressed the expression of stem cell markers including DCLK1, CD133, BMI1, and CD44v9. *p < 0.05. **p < 0.001. Data presented as mean ± SD. (D) qRT‐PCR showed that treatment with Tg significantly increased the expression of the epithelial differentiation marker, CDX2. *p < 0.05. Data presented as mean ± SD

To explore the molecular basis of the effects of silencing LINC01534 in CRC cells, we performed RNA sequencing. Gene ontology analysis indicated that silencing LINC01534 led to the enrichment of multiple gene sets associated with the ER stress response (Figure S5A). Among three ER stress sensors (PERK, IRE1α, and ATF6α), expression of PERK and the molecules acting at downstream pathway was upregulated (Figure S5B). Thus, LINC01534‐silenced HCT116 cells exhibited increased RNA expression of the chaperone binding‐immunoglobulin protein (BiP) (encoded by HSPA5) (2.87‐fold), PERK (1.94‐fold), ATF4 (1.36‐fold), and CCAAT/enhancer‐binding protein homologous protein (CHOP) (4.31‐fold) as compared with the parent cells (Figure S5B). To confirm these preliminary data, we used western blot analysis to examine phosphorylation of eIF2α, which is a hallmark of PERK pathway activation. Compared with the negative control siRNA treatment, silencing LINC01534 in HCT116 cells resulted in upregulation of phosphorylated eIF2α, as well as PERK and ATF4, 9 h after transfection (Figure 3A). In RKO cells, a marked increase in phosphorylated eIF2α as well as PERK and ATF4 was found by siLINC01534 treatment at the later timepoint of 24 h (Figure 3B). In glucose‐ or glutamine‐deprived cultures of both CRC cell lines, LINC01534 silencing resulted in more pronounced upregulation of the PERK/eIF2α signaling pathway‐related molecules, PERK, HSPA5, ATF4, and phosphorylated eIF2α (Figure 3A,B).

Treatment with the unfolded protein response (UPR) inducer thapsigargin resulted in significant repression of stem cell markers including DCLK1, CD133, BMI1, and CD44v9, and led to a significant increase of the epithelial differentiation marker, CDX2 in HCT116 cells (*p < 0.05, **p < 0.001, Figure 3C,D), indicating that ER stress response suppresses cancer stemness.

### 3.7 Localization of LINC01534 in colonic normal mucosa and tumor tissue

**FIGURE 4:**
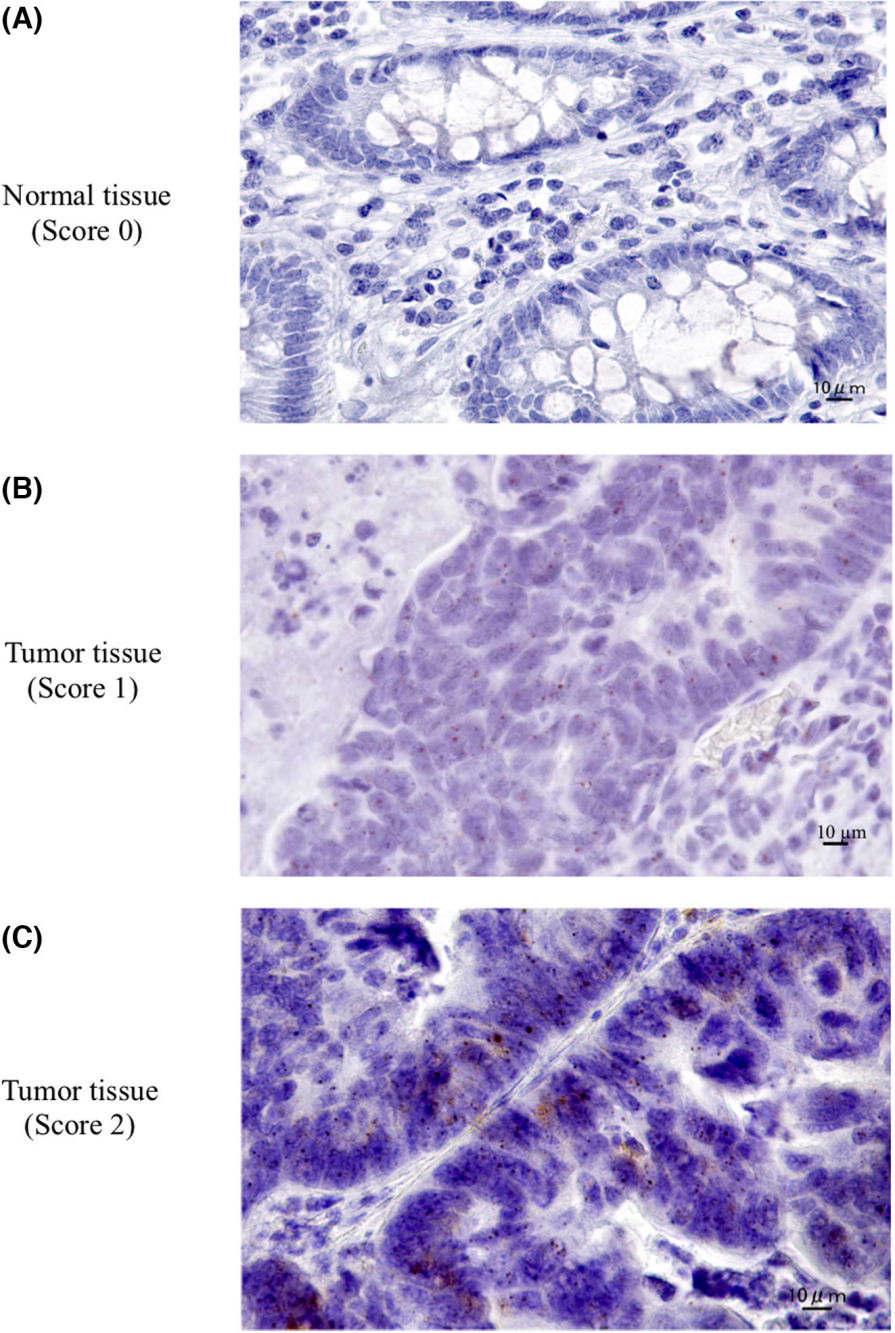
In situ hybridization using RNAscope in normal epithelium and CRC tissues. (A) In normal mucosa, LINC01534 expression was generally not detected (score 0). (B) In a tumor tissue, LINC01534 expression was noted mainly in tumor cells. The LINC01534 signal was 1–3 dots/cell (score 1). (C) In another tumor tissue, increased expression of LINC01534 was found mainly in tumor cells. The LINC01534 signal was 4–9 dots/cell (score 2). Scale bar, 10 μm

In situ hybridization for LINC01534 was performed using RNAscope in 12 paired normal and CRC tumor tissue samples. LINC01534 was not detected in normal colonic epithelium (Figure 4A). LINC01534 expression was detected in tumor tissues, and was localized in epithelial tumor cells but not at the tumor stroma (Figure 4B,C). When the LINC01534 expression level in CRC tissue was classified into four levels (scores 0–3), as described in METHODS, five CRC tissue samples were classified as score 1, and seven samples as score 2. By contrast, normal tissue samples were all classified as score 0.

### 3.8 LINC01534 expression is associated with poor prognosis of CRC patients

**FIGURE 5:**
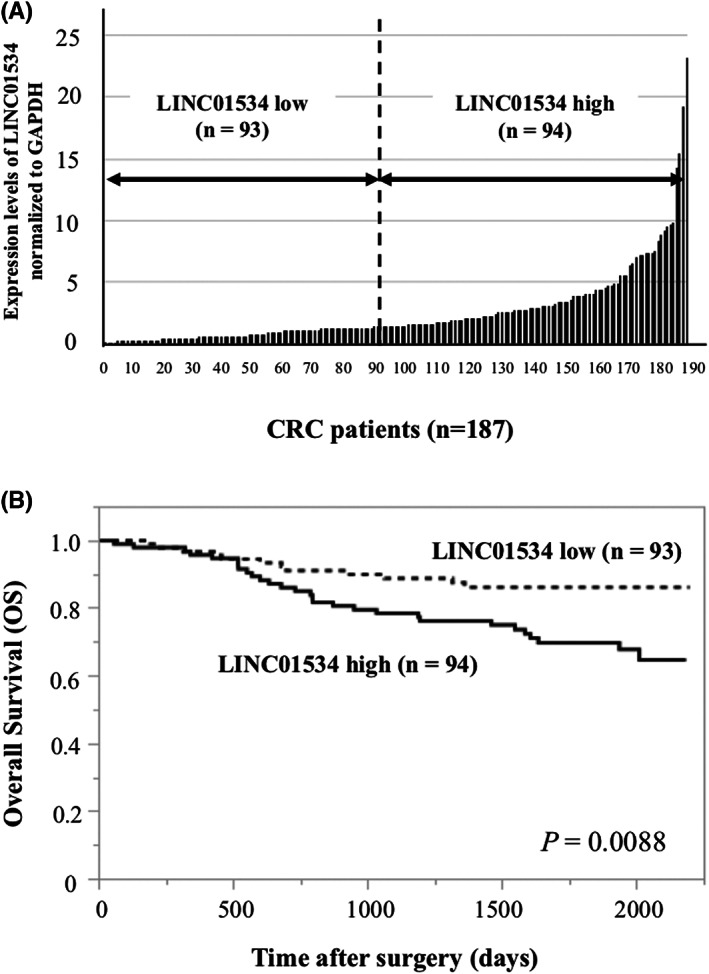
Association of LINC01534 expression with overall survival (OS) of CRC patients. (A) LINC01534 levels in 187 CRC tissue samples. LINC01534 expression in the CRC tissue sample was measured by qRT‐PCR and GAPDH RNA expression was used for normalization. The patients were divided into high‐ and low‐expression groups, based on the median LINC01534 value of 1.53. (B) Kaplan–Meier OS curves according to the LINC01534 level. The OS rate was significantly lower in the LINC01534 high‐expression group (*n* = 94) compared with the low‐expression group (*n* = 93; log‐rank test; *p =* 0.0088)

As suggested by the TCGA dataset we tried to clarify the significance of LINC01534 RNA expression in the OS of the CRC patients who underwent surgery at Osaka University and its related hospitals. One hundred eighty‐seven patients with CRC were divided into a LINC01534 high‐expression group (*n* = 94) and a low‐expression group (*n* = 93), based on the median LINC01534 value of 1.53 in tumor tissue (Figure 5A). As for the relationship between clinicopathologic characteristics and LINC01534 expression, high LINC01534 expression was significantly associated with Stage IV CRC (*p =* 0.025, Table 1). With regard to OS, patients with high LINC01534 expression had a significantly poorer prognosis than patients with low LINC01534 expression (*p =* 0.0088; Figure 5B). Univariate analysis showed that tumor depth, lymph node metastasis, lymphatic invasion, venous invasion, and LINC01534 expression were significant prognostic factors for OS (Table 2). Multivariate analysis revealed that LINC01534 expression was an independent prognostic factor for OS (*p =* 0.047) as well as for T4 tumors and the presence of lymphatic invasion (Table 2).

**TABLE 1:** LINC01534 expression and clinicopathological factors

Variables	LINC01534 expression		*p* value
	Low (*n* = 93)	High (*n* = 94)	
Sex (male/female)	54/39	53/41	0.817
Tumor location (right/left)	32/61	27/67	0.403
Tumor size, cm, median [range]	6.0 [1.5–8.2]	5.0 [1.6–10]	0.121
Tumor depth (pT0‐3/pT4)	49/44	44/50	0.421
Lymph node metastasis positive (yes/no)	44/49	53/41	0.215
^*^Histologic grade (muc or por or sig/tub1,2)	5/88	3/91	0.497
Lymphatic invasion positive (yes/no)	62/31	68/26	0.400
Venous invasion positive (yes/no)	54/39	64/30	0.156
pStage (I‐III/IV)	82/11	71/23	0.025^a^

^*^ muc, mucinous adenocarcinoma; por, poorly differentiated adenocarcinoma; sig, signet‐ring cell carcinoma; tub, tubular adenocarcinoma.

^a^ *p <* 0.05.

**TABLE 2:** Univariate and multivariate analyses for overall survival

Variables	Number	Univariate analysis		Multivariate analysis	
		Relative risk	*p* value	Relative risk	*p* value
Sex (male/female)	107/80	0.72	0.297		
Tumor location (right/left)	59/128	1.41	0.294		
Tumor depth (pT0‐3/pT4)	93/94	3.97	0.0003^a^	2.52	0.017^a^
Lymph node metastasis (yes/no)	97/90	3.18	0.002^a^	2.01	0.062
^*^Histologic grade (muc or por or sig/tub1, 2)	8/179	2.10	0.217		
Lymphatic invasion (yes/no)	130/57	6.02	0.003^a^	3.69	0.0034^a^
Venous invasion (yes/no)	118/69	3.76	0.003^a^	1.86	0.178
LINC01534 expression (high/low)	94/93	2.39	0.011^a^	1.99	0.047^a^

^*^ muc, mucinous adenocarcinoma; por, poorly differentiated adenocarcinoma; sig, signet‐ring cell carcinoma; tub, tubular adenocarcinoma.

^a^ *p* < 0.05.

## 4 DISCUSSION

In this study we found by lncRNA sequencing that LINC01534 was highly expressed in ODC degron transduced HCT116 cells. The TCGA dataset indicated that only the two lncRNAs, LINC01534 and HOTAIR, were significantly associated with shorter OS of CRC patients among the lncRNAs listed in Figure S1A. HOTAIR is known as an oncogenic lncRNA in various type of human cancer.^8^ However, the LINC01534 literature was limited solely to the inflammatory disease osteoarthritis^10^ and we are not aware of any reports on LINC01534 in human cancer.

In vitro analyses using CRC cell lines revealed that inhibition of LINC01534 induced apoptosis and suppressed cell proliferation, cell cycle progression, invasiveness, and cancer stemness. We also demonstrated that high LINC01534 expression was associated with stage IV CRC, the presence of metastasis, and poor OS in CRC patients. To our knowledge, this is the first study to explore a crucial role of LINC01534 in CRC.

We previously demonstrated that LPACs, including HCT116, have cancer stem‐like properties, such as high sphere formation ability, tumor growth, and drug resistance, by using the ZsGreen‐ODC degron system.^11^ Studies have shown that inhibition of proteasome activity led to activation of ER stress response, namely UPR.^26^, ^27^, ^28^ This appears reasonable because the misfolded proteins could accumulate in the cells under the low proteasome condition, which triggers on the UPR. Consistently, we found that LPACs in HCT116 displayed increased phosphorylation of translation initiation factor eIF2α as the indictor of UPR activation,^14^ under cultures supplemented either with a standard medium or a glutamine‐deprivation medium, as compared with non‐LPACs (Figure S6). We also confirmed that the phosphorylated eIF2α level increased when the proteasome inhibitor lactacystin was administered to the parental HCT116 cells (Figure S7).

As for the effect of LINC01534 inhibition in proteasome activity in LPACs of HCT116, no significant effect was noted (Figure S8A), but treatment with siLINC01534 still inhibited the expression of the CSC markers, DCLK1 and Bmi1 (Figure S8B). It is therefore supposed that upregulation of LINC01534 in LPACs might suppress further activation of ER stress response to maintain cancer stemness. In any case, LPAC is just a model for CSC with low proteasome activity. Thus, it would be better, irrespective of proteasome activity, to explore the role of LINC01534 in the parental HCT116 cells, which is known to retain cancer stem cell properties.^21^, ^22^, ^23^

We found that siRNA treatment for LINC01534 resulted in reduced sphere formation and decreased expression of a series of CSC markers in HCT116 cells. These findings suggest that LINC01534 plays an essential role in maintaining cancer stemness. This notion is further supported by the findings that siRNA treatment induced an epithelial differentiation marker CDX2 and enhanced the sensitivity of HCT116 cells to 5‐FU.

One of the major findings in this study is that LINC01534 appeared to suppress the ER stress response. ER stress is a condition in which unfolded or incorrect proteins accumulate in the ER due to various cellular stresses; for example, nutrient deprivation, hypoxia, oxidative stress, and anticancer drug treatment.^14^, ^16^, ^29^ Under normal physiological conditions, the chaperone protein HSPA5 binds to three ER stress sensors (ER‐spanning transmembrane proteins), such as PERK, activating transcription factor 6α (ATF6α), and inositol‐requiring enzyme 1α (IRE1α), which remain sensors in their monomeric inactive states.^14^, ^29^ Upon exposure to ER stress, HSPA5 is released from the sensors and binds to unfolded proteins, starting the UPR.^29^ Activated PERK then phosphorylates eIF2α, which restricts mRNA translation and thereby reduces the influx of nascent proteins into the ER.^14^, ^30^ Through this process, ER stress can be reduced and the cells can restore ER homeostasis through the UPR.^14^, ^15^, ^29^ A moderate level of UPR enhances cancer cell survival, angiogenesis, metastatic capacity, drug resistance, and immunosuppression,^4^, ^14^, ^31^ while excessive or prolonged activation of the UPR may lead to apoptotic cell death.^15^, ^29^, ^31^

Upon inhibition of LINC01534, the ER stress response via the PERK/eIF2α signaling pathway was activated in CRC cells. This activation by siLINC01534 treatment was more clearly demonstrated under glucose‐ and glutamine‐deprivation conditions, both of which induce potent ER stress. These findings suggest that LINC01534 likely plays a role in suppressing the ER stress response in CRC cells.

One may suppose that alteration of the two phenomena, cancer stemness and the ER stress response caused by LINC01534, may not be related to each other. However, the two aspects are tightly linked in CRC. Thus, it has been demonstrated that cancer stemness and the ER stress response are inversely regulated in CRC.^12^, ^13^ ER stress is reported to cause the loss of intestinal epithelial stemness through activation of a UPR.^12^ Moreover, activation of the UPR reportedly forces colon‐CSCs to differentiate, which augments their sensitivity to chemotherapy.^13^ These findings suggest that the UPR should be negatively regulated to maintain CSC properties. We also confirmed that thapsigargin, an inducer of UPR, indeed suppressed the expression of cancer stem markers and instead enhanced the expression of the differentiation marker CDX2. In this regard, we postulate that LINC01534 plays a central role in the maintenance of cancer stemness through suppression of the UPR.

Cell cycle analysis revealed that siRNA treatment against LINC01534 caused G2‐M arrest in CRC cells. Consistently, western blot analysis revealed decreased levels of G2‐M accelerators, inducing cdc25B, cdc25C, and cdc2. Studies have described ER stress‐mediated cell cycle arrest at G2‐M being regulated through the PERK/eIF2α signaling pathway in relation to modulation of a G2‐M acting cell cycle component, either cdc2 or cdc25C.^15^, ^16^, ^30^ Lee et al reported that ER stress and subsequent UPR induction led to arrest of cell cycle progression at the G2‐M phase via elF2α phosphorylation.^30^ It has also been shown that the polyphenol derivative butein induces the ER stress response by activating the PERK/eIF2α signaling pathway, while simultaneously inducing G2‐M arrest.^15^ Therefore, it is assumed that the G2‐M arrest caused by siLINC01534 treatment might be attributed to activation of the ER stress response.

Our data indicate that high expression of LINC01534 was associated with metastasis and poor prognosis of CRC patients. This could, in part, be attributed to its role in the maintenance of cancer stemness that we demonstrated. On the other hand, it remains controversial how the ER stress response influences tumor dissemination and metastasis.^31^, ^32^ ER stress in cancer cells was recently shown to modulate the tumor immune microenvironment.^31^ Notably, in a recent study Pommier et al demonstrated that unresolved ER stress enables disseminated pancreatic cancer cells to escape immunity and establish latent metastases.^32^ Thus, we postulate that LINC01534 preserves unsolved ER stress by suppressing UPR, and such unfolded proteins may protect disseminated CRC cells from T‐cell immunity, thus leading to distant metastasis. However, further investigations are needed to uncover the mechanisms connecting LINC01534 and immunity.

In conclusion, we demonstrated for the first time that LINC01534 is a novel prognostic factor in CRC. LINC01534 facilitates the proliferation and invasiveness of CRC cells and has a unique function in the maintenance of cancer stemness and coordinated suppression of the ER stress response. Targeting LINC01534 is a potential therapeutic option against CRC that may improve the outcome in CRC patients.

## AUTHOR CONTRIBUTIONS

MI, HT, MO, and HY provided conception and design of the study, and contributed to acquisition and analysis of data. NN, MM, YD, and HE provided conception and design of the study. CI, DO, YY, NM, MU, GAC, and ST contributed to acquisition and analysis of data. MI, NN, and HY wrote the article. All authors were engaged in interpretation of data, revising the article, approved the final version of article, and agreed in all aspects of the work. ST and HY performed ODC degron‐related experiments. CI and GAC provided TCGA data and performed survival analysis. HT, NM, MU, YD, and HE collected and provided clinical samples and data. DO worked in RNA sequencing and analysis.

## FUNDING INFORMATION

This study was partly supported by a research grant from JSPS KAKENHI (21 K08733) and Kagoshima Shinsangyo Sousei Investment Limited Partnership (its general partner is Kagoshima Development Co., Ltd).

## CONFLICT OF INTEREST

The authors have no conflict of interest to disclose. The author M.M. is Emeritus Editor‐in‐Chief of *Annals of Gastroenterological Surgery*. The authors S.T. and Y.D. were editorial members of *Annals of Gastroenterological Surgery*.

## ETHICS STATEMENT

Approval of the Research Protocol: This study protocol was approved by the Institutional Review Board of Osaka University Hospital (No. 15144). Informed Consent: Informed consent was obtained from all patients before surgery. Registry and the Registration: This research was not preregistered in an independent, institutional registry (N/A). Animal Studies: This research had no animal studies (N/A).

REFERENCES1

Bray
F
, 
Ferlay
J
, 
Soerjomataram
I
, 
Siegel
RL
, 
Torre
LA
, 
Jemal
A
. Global cancer statistics 2018: GLOBOCAN estimates of incidence and mortality worldwide for 36 cancers in 185 countries. CA Cancer J Clin. 2018;68(6):394–424.3020759310.3322/caac.214922

Colvin
H
, 
Mizushima
T
, 
Eguchi
H
, 
Takiguchi
S
, 
Doki
Y
, 
Mori
M
. Gastroenterological surgery in Japan: the past, the present and the future. Ann Gastroenterol Surg. 2017;1(1):5–10.2986312910.1002/ags3.12008PMC58812963

Brenner
H
, 
Kloor
M
, 
Pox
CP
. Colorectal cancer. Lancet. 2014;383(9927):1490–502.2422500110.1016/S0140-6736(13)61649-94

Tang
GH
, 
Chen
X
, 
Ding
JC
, 
Du
J
, 
Lin
XT
, 
Xia
L
, et al. LncRNA LUCRC regulates colorectal cancer cell growth and tumorigenesis by targeting endoplasmic reticulum stress response. Front Genet. 2020;10:1409.3208236510.3389/fgene.2019.01409PMC70052515

Kopp
F
, 
Mendell
JT
. Functional classification and experimental dissection of long noncoding RNAs. Cell. 2018;172(3):393–407.2937382810.1016/j.cell.2018.01.011PMC59787446

Guttman
M
, 
Rinn
JL
. Modular regulatory principles of large non‐coding RNAs. Nature. 2012;482(7385):339–46.2233705310.1038/nature10887PMC41970037

Hon
CC
, 
Ramilowski
JA
, 
Harshbarger
J
, 
Bertin
N
, 
Rackham
OJL
, 
Gough
J
, et al. An atlas of human long non‐coding RNAs with accurate 5′ ends. Nature. 2017;543(7644):199–204.2824113510.1038/nature21374PMC68571828

Gupta
RA
, 
Shah
N
, 
Wang
KC
, 
Kim
J
, 
Horlings
HM
, 
Wong
DJ
, et al. Long non‐coding RNA HOTAIR reprograms chromatin state to promote cancer metastasis. Nature. 2010;464(7291):1071–6.2039356610.1038/nature08975PMC30499199

Raveh
E
, 
Matouk
IJ
, 
Gilon
M
, 
Hochberg
A
. The H19 long non‐coding RNA in cancer initiation, progression and metastasis ‐ a proposed unifying theory. Mol Cancer. 2015;14:184.2653686410.1186/s12943-015-0458-2PMC463268810

Wei
W
, 
He
S
, 
Wang
Z
, 
Dong
J
, 
Xiang
D
, 
Li
Y
, et al. LINC01534 promotes the aberrant metabolic dysfunction and inflammation in IL‐1β‐simulated osteoarthritic chondrocytes by targeting miR‐140‐5p. Cartilage. 2021;13:898 S–907 S.10.1177/1947603519888787PMC88047873173507711

Munakata
K
, 
Uemura
M
, 
Tanaka
S
, 
Kawai
K
, 
Kitahara
T
, 
Miyo
M
, et al. Cancer stem‐like properties in colorectal cancer cells with low proteasome activity. Clin Cancer Res. 2016;22(21):5277–86.2716639510.1158/1078-0432.CCR-15-194512

Heijmans
J
, 
van Lidth de Jeude
JF
, 
Koo
BK
, 
Rosekrans
SL
, 
Wielenga
MC
, 
van de Wetering
M
, et al. ER stress causes rapid loss of intestinal epithelial stemness through activation of the unfolded protein response. Cell Rep. 2013;3(4):1128–39.2354549610.1016/j.celrep.2013.02.03113

Wielenga
MCB
, 
Colak
S
, 
Heijmans
J
, 
van Lidth de Jeude
JF
, 
Rodermond
HM
, 
Paton
JC
, et al. ER‐stress‐induced differentiation sensitizes colon cancer stem cells to chemotherapy. Cell Rep. 2015;13(3):489–94.2645682410.1016/j.celrep.2015.09.01614

Cubillos‐Ruiz
JR
, 
Bettigole
SE
, 
Glimcher
LH
. Tumorigenic and immunosuppressive effects of endoplasmic reticulum stress in cancer. Cell. 2017;168(4):692–706.2818728910.1016/j.cell.2016.12.004PMC533375915

Di
S
, 
Fan
C
, 
Ma
Z
, 
Li
M
, 
Guo
K
, 
Han
D
, et al. PERK/eIF‐2α/CHOP pathway dependent ROS generation mediates Butein‐induced non‐small‐cell lung cancer apoptosis and G2/M phase arrest. Int J Biol Sci. 2019;15(8):1637–53.3136010710.7150/ijbs.33790PMC664321516

Xia
D
, 
Ji
W
, 
Xu
C
, 
Lin
X
, 
Wang
X
, 
Xia
Y
, et al. Knockout of MARCH2 inhibits the growth of HCT116 colon cancer cells by inducing endoplasmic reticulum stress. Cell Death Dis. 2017;8(7):e2957.2874946610.1038/cddis.2017.347PMC558461517

Adikrisna
R
, 
Tanaka
S
, 
Muramatsu
S
, 
Aihara
A
, 
Ban
D
, 
Ochiai
T
, et al. Identification of pancreatic cancer stem cells and selective toxicity of chemotherapeutic agents. Gastroenterology. 2012;143(1):234–45.2251020210.1053/j.gastro.2012.03.05418

Makino
S
, 
Takahashi
H
, 
Okuzaki
D
, 
Miyoshi
N
, 
Haraguchi
N
, 
Hata
T
, et al. DCLK1 integrates induction of TRIB3, EMT, drug resistance and poor prognosis in colorectal cancer. Carcinogenesis. 2020;41(3):303–12.3156274110.1093/carcin/bgz15719

Sugimura
K
, 
Fujiwara
Y
, 
Omori
T
, 
Motoori
M
, 
Miyoshi
N
, 
Akita
H
, et al. Clinical importance of a transcription reverse‐transcription concerted (TRC) diagnosis using peritoneal lavage fluids obtained pre‐ and post‐lymphadenectomy from gastric cancer patients. Surg Today. 2016;46(6):654–60.2627248610.1007/s00595-015-1235-y20

Yukimoto
R
, 
Nishida
N
, 
Hata
T
, 
Fujino
S
, 
Ogino
T
, 
Miyoshi
N
, et al. Specific activation of glycolytic enzyme enolase 2 in BRAF V600E‐mutated colorectal cancer. Cancer Sci. 2021;112(7):2884–94.3393442810.1111/cas.14929PMC825329021

Sadanandam
A
, 
Lyssiotis
CA
, 
Homicsko
K
, 
Collisson
EA
, 
Gibb
WJ
, 
Wullschleger
S
, et al. A colorectal cancer classification system that associates cellular phenotype and responses to therapy. Nat Med. 2013;19(5):619–25.2358408910.1038/nm.3175PMC377460722

Yeung
TM
, 
Gandhi
SC
, 
Wilding
JL
, 
Muschel
R
, 
Bodmer
WF
. Cancer stem cells from colorectal cancer‐derived cell lines. Proc Natl Acad Sci U S A. 2010;107(8):3722–7.2013359110.1073/pnas.0915135107PMC284041623

Chen
KL
, 
Pan
F
, 
Jiang
H
, 
Chen
JF
, 
Pei
L
, 
Xie
FW
, et al. Highly enriched CD133(+)CD44(+) stem‐like cells with CD133(+)CD44(high) metastatic subset in HCT116 colon cancer cells. Clin Exp Metastasis. 2011;28(8):751–63.2175090710.1007/s10585-011-9407-724

Das
U
, 
Alcorn
J
, 
Shrivastav
A
, 
Sharma
RK
, 
Clercq
ED
, 
Balzarini
J
, et al. Design, synthesis and cytotoxic properties of novel 1‐[4‐(2‐alkylaminoethoxy)phenylcarbonyl]‐3,5‐bis(arylidene)‐4‐piperidones and related compounds. Eur J Med Chem. 2007;42(1):71–80.1699665710.1016/j.ejmech.2006.08.00225

Kraljević
TG
, 
Kristafor
S
, 
Suman
L
, 
Kralj
M
, 
Ametamey
SM
, 
Cetina
M
, et al. Synthesis, X‐ray crystal structure study and antitumoral evaluations of 5,6‐disubstituted pyrimidine derivatives. Bioorg Med Chem. 2010;18(7):2704–12.2021156410.1016/j.bmc.2010.02.02326

Obeng
EA
, 
Carlson
LM
, 
Gutman
DM
, 
Harrington
WJ
Jr
, 
Lee
KP
, 
Boise
LH
. Proteasome inhibitors induce a terminal unfolded protein response in multiple myeloma cells. Blood. 2006;107(12):4907–16.1650777110.1182/blood-2005-08-3531PMC189581727

Ri
M
. Endoplasmic‐reticulum stress pathway‐associated mechanisms of action of proteasome inhibitors in multiple myeloma. Int J Hematol. 2016;104(3):273–80.2716961410.1007/s12185-016-2016-028

Oakes
SA
. Endoplasmic reticulum stress signaling in cancer cells. Am J Pathol. 2020;190(5):934–46.3211271910.1016/j.ajpath.2020.01.010PMC723782929

Hetz
C
, 
Papa
FR
. The unfolded protein response and cell fate control. Mol Cell. 2018;69(2):169–81.2910753610.1016/j.molcel.2017.06.01730

Lee
D
, 
Hokinson
D
, 
Park
S
, 
Elvira
R
, 
Kusuma
F
, 
Lee
JM
, et al. ER stress induces cell cycle arrest at the G2/M phase through eIF2α phosphorylation and GADD45α. Int J Mol Sci. 2019;20(24):6309.3184723410.3390/ijms20246309PMC694079331

Chen
X
, 
Cubillos‐Ruiz
JR
. Endoplasmic reticulum stress signals in the tumour and its microenvironment. Nat Rev Cancer. 2021;21(2):71–88.3321469210.1038/s41568-020-00312-2PMC792788232

Pommier
A
, 
Anaparthy
N
, 
Memos
N
, 
Kelley
ZL
, 
Gouronnec
A
, 
Yan
R
, et al. Unresolved endoplasmic reticulum stress engenders immune‐resistant, latent pancreatic cancer metastases. Science. 2018;360(6394):e4908.10.1126/science.aao4908PMC654738029773669

## Supporting information


Figure S1

Figure S2

Figure S3

Figure S4

Figure S5

Figure S6

Figure S7

Figure S8

Table S1

Table S2

## Data Availability

Access to raw data concerning this study was submitted under Gene Expression Omnibus (https://www.ncbi.nlm.nih.gov/geo/query/acc.cgi?acc=GSE179624 , enter ilktygmgdpglxex into the box). For the survival analysis of lncRNA expression is listed in Figure S1B, The Cancer Genome Atlas Program (TCGA) database was used (https://www.cancer.gov/about‐nci/organization/ccg/research/structural‐genomics/tcga/using‐tcga).
