# Supplementary material for: Long noncoding RNA 01534 maintains cancer stemness by downregulating endoplasmic reticulum stress response in colorectal cancer
Source: Ann Gastroenterol Surg. 2022 Dec 29;7(3):458–70. doi: 10.1002/ags3.12649 (PMC10154865; doi:10.1002/ags3.12649)
Supplement: Supplementary file 1 — Figure S1 [file AGS3-7-458-s009.pdf]

Supplemental figure 1

A

LncRNA	Fold change	*P* value
ARMCX3-AS1	21.934	0.020
RP11-396F22.1	16.301	0.035
HOTAIR	15.999	0.046
OXCT1-AS1	7.025	0.040
CH507-513H4.5	6.805	0.042
AC004019.13	6.058	0.044
RP11-482H16.1	5.216	0.009
CTD-2033D15.2	4.791	0.000
RP11-234O6.2	4.184	0.015
RP11-91I20.4	4.111	0.003
RP11-298I3.4	4.042	0.019
CTD-2265O21.7	3.877	0.042
ENTPD3-AS1	3.792	0.048
AC016999.2	3.778	0.043
RP11-452H21.4	3.269	0.034
RP11-1379J22.2	3.179	0.004
RP1-224A6.9	3.019	0.011
RP11-61F12.1	2.786	0.007
**LINC01534**	2.767	0.019
CTD-2619J13.8	2.669	0.023
RDH10-AS1	2.651	0.019
POT1-AS1	2.489	0.019
-	2.476	0.049
RP11-844P9.5	2.437	0.006
CTD-2649C14.2	2.432	0.044
RP11-119F7.5	2.401	0.017
AC133644.2	2.305	0.005
PRKG1-AS1	2.294	0.022
AC005104.3	2.293	0.038
SCARNA15	2.234	0.000
CTD-2538C1.2	2.162	0.013
RP11-620J15.3	2.080	0.004
RP11-264B17.2	2.017	0.019
RP11-351C21.2	2.005	0.029

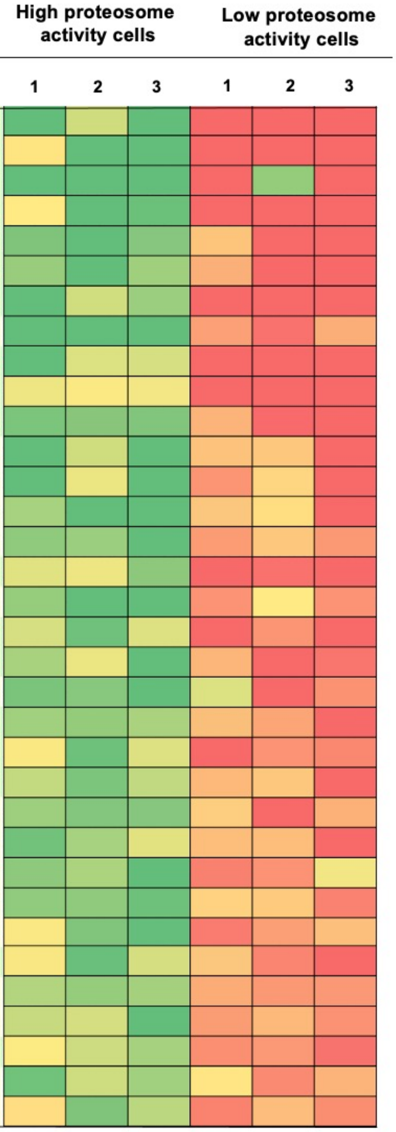

B

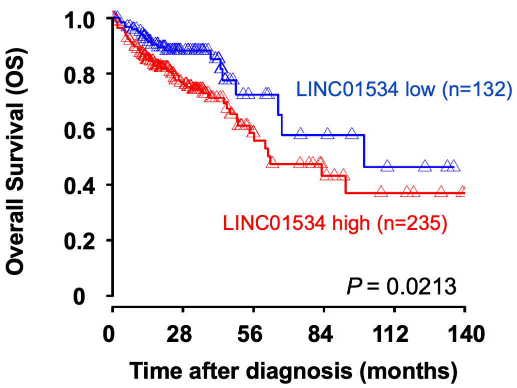

Supplemental figure 1.

A. Comprehensive transcriptome analysis by lncRNA sequencing comparing low proteasome activity cells (LPACs) and non-LPACs in HCT116 cells. Cells in each group were collected in triplicates and differential expression of lncRNAs were analyzed. Because LPACs tended to return non-LPACs, they were collected in Trizol solution just after sorting by the Cell Sorter SH800Z. Thirty-four lncRNAs were up-regulated at >2.0 folds in LAPCs as compared with non-LPACs.

B. TCGA dataset indicated that high expression of LINC01534 was significantly associated with shorter overall survival. The high LINC01534 expression group showed significantly lower overall survival than the low expression group (cut-off = 0.36; log-rank test; $P = 0.0213$).
