# Supplementary figures and images for: Long noncoding RNA 01534 maintains cancer stemness by downregulating endoplasmic reticulum stress response in colorectal cancer
Source: Ann Gastroenterol Surg. 2022 Dec 29;7(3):458–70. doi: 10.1002/ags3.12649 (PMC10154865; doi:10.1002/ags3.12649)

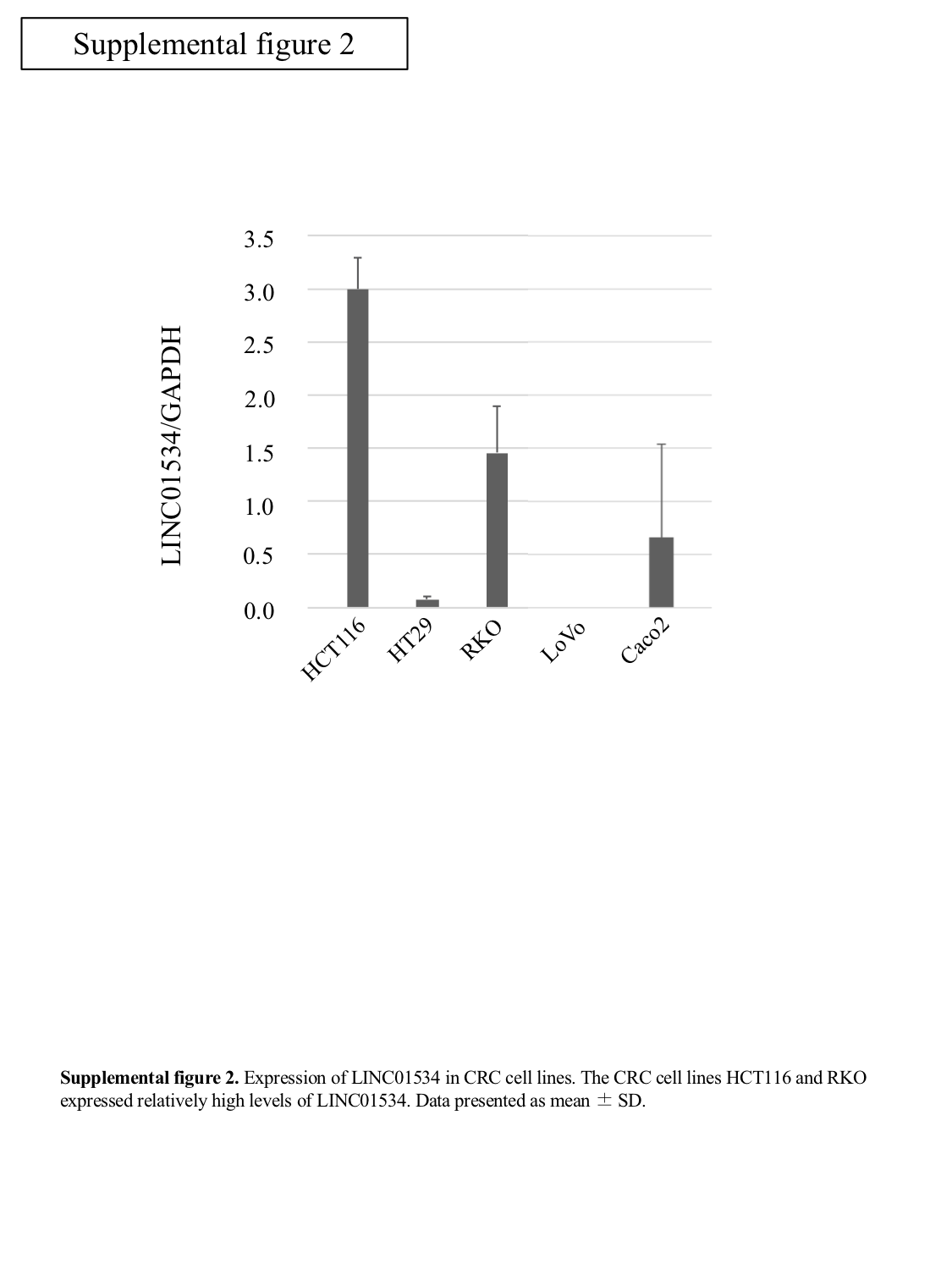

Supplement: Supplementary file 2 — Figure S2 [file AGS3-7-458-s004.tiff]

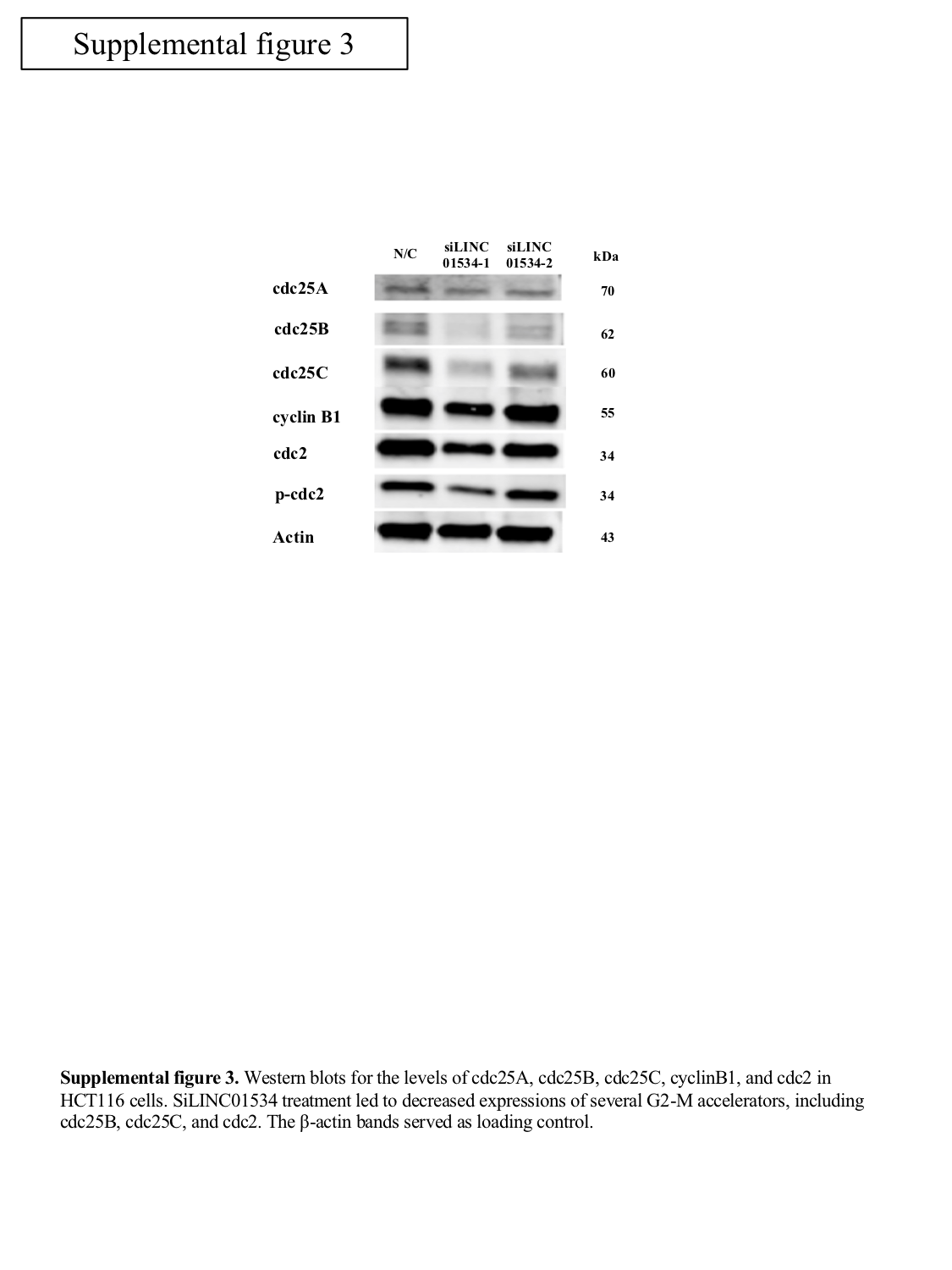

Supplement: Supplementary file 3 — Figure S3 [file AGS3-7-458-s008.tiff]

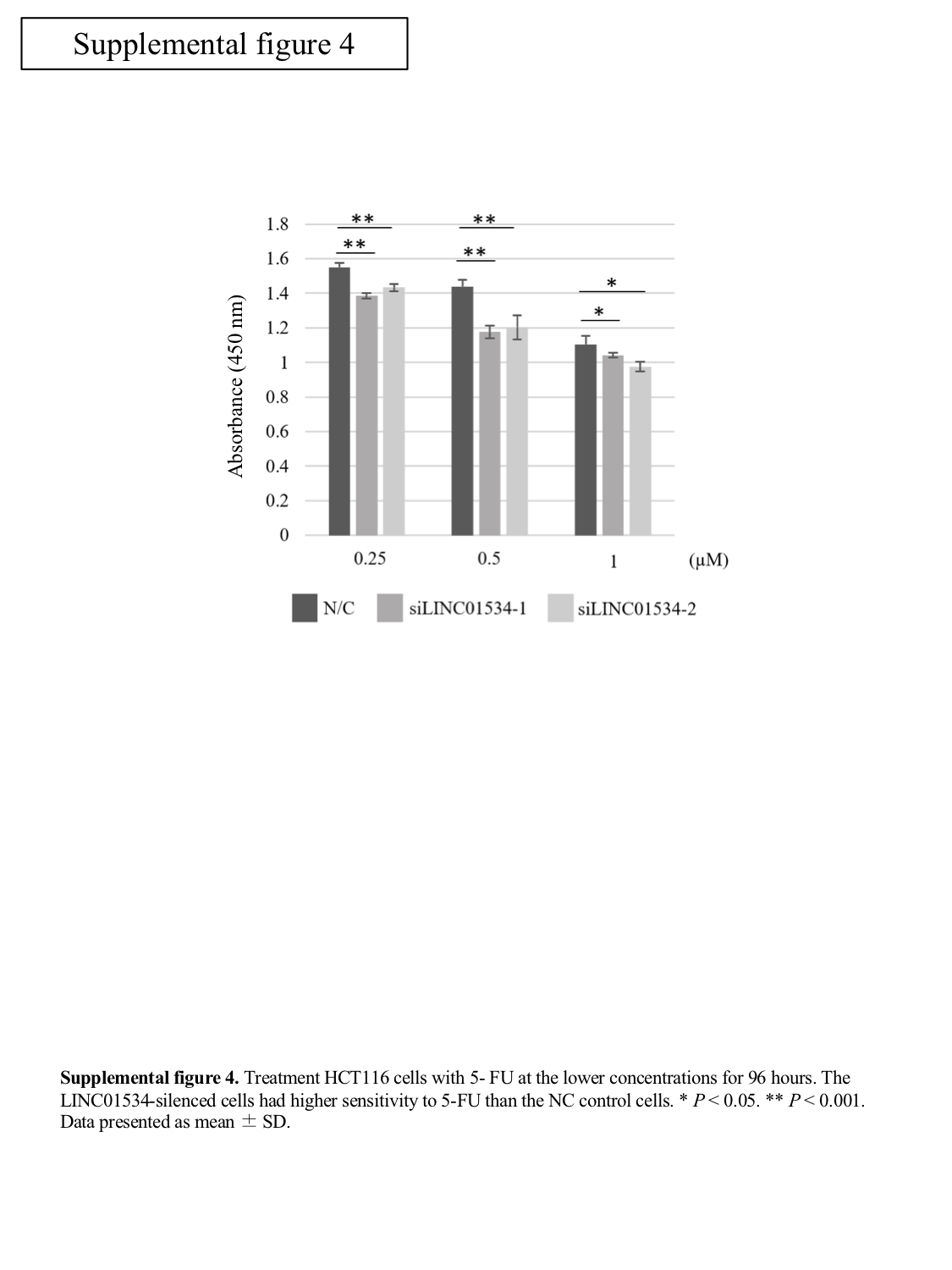

Supplement: Supplementary file 4 — Figure S4 [file AGS3-7-458-s003.tiff]

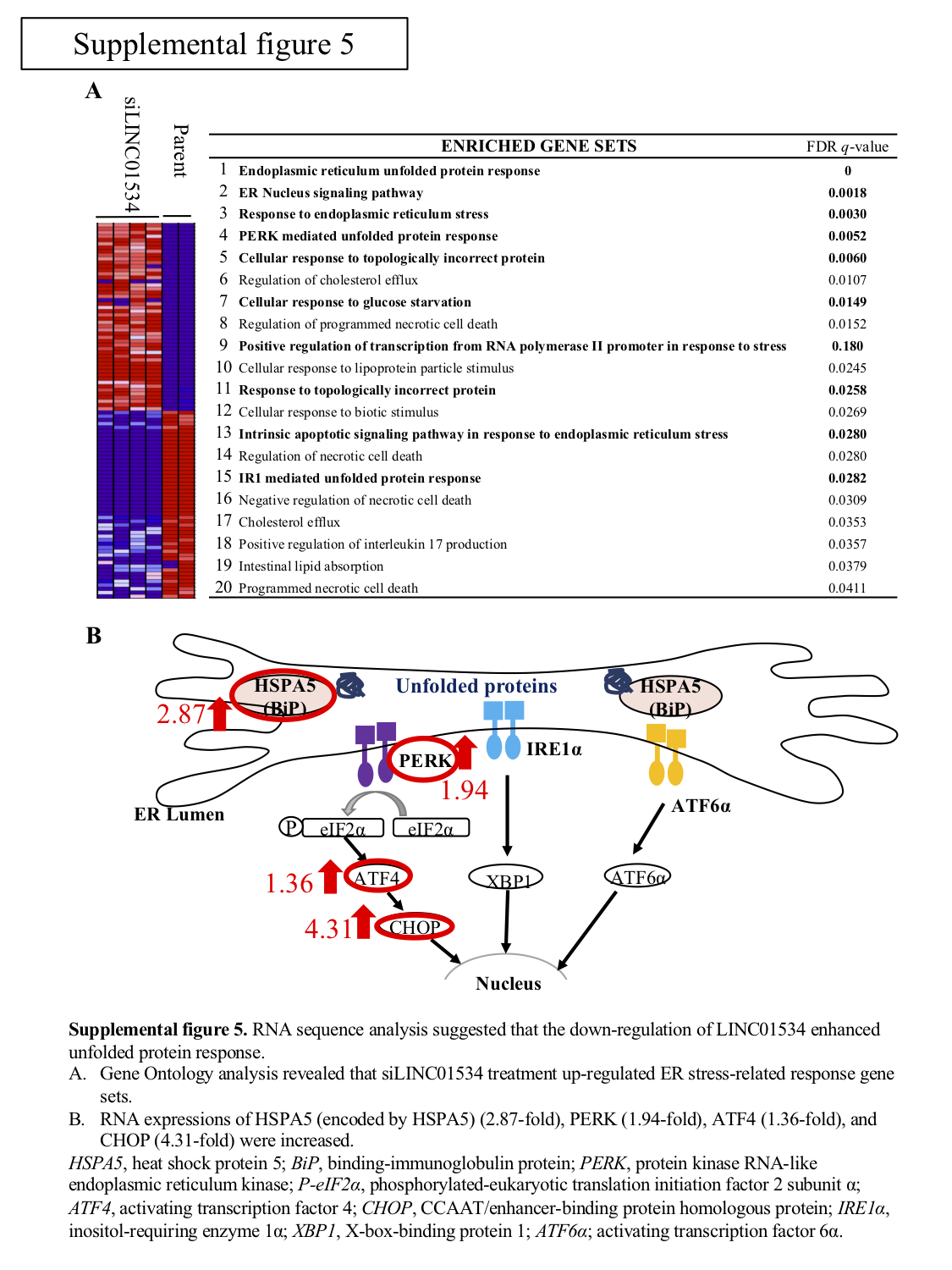

Supplement: Supplementary file 5 — Figure S5 [file AGS3-7-458-s005.tiff]

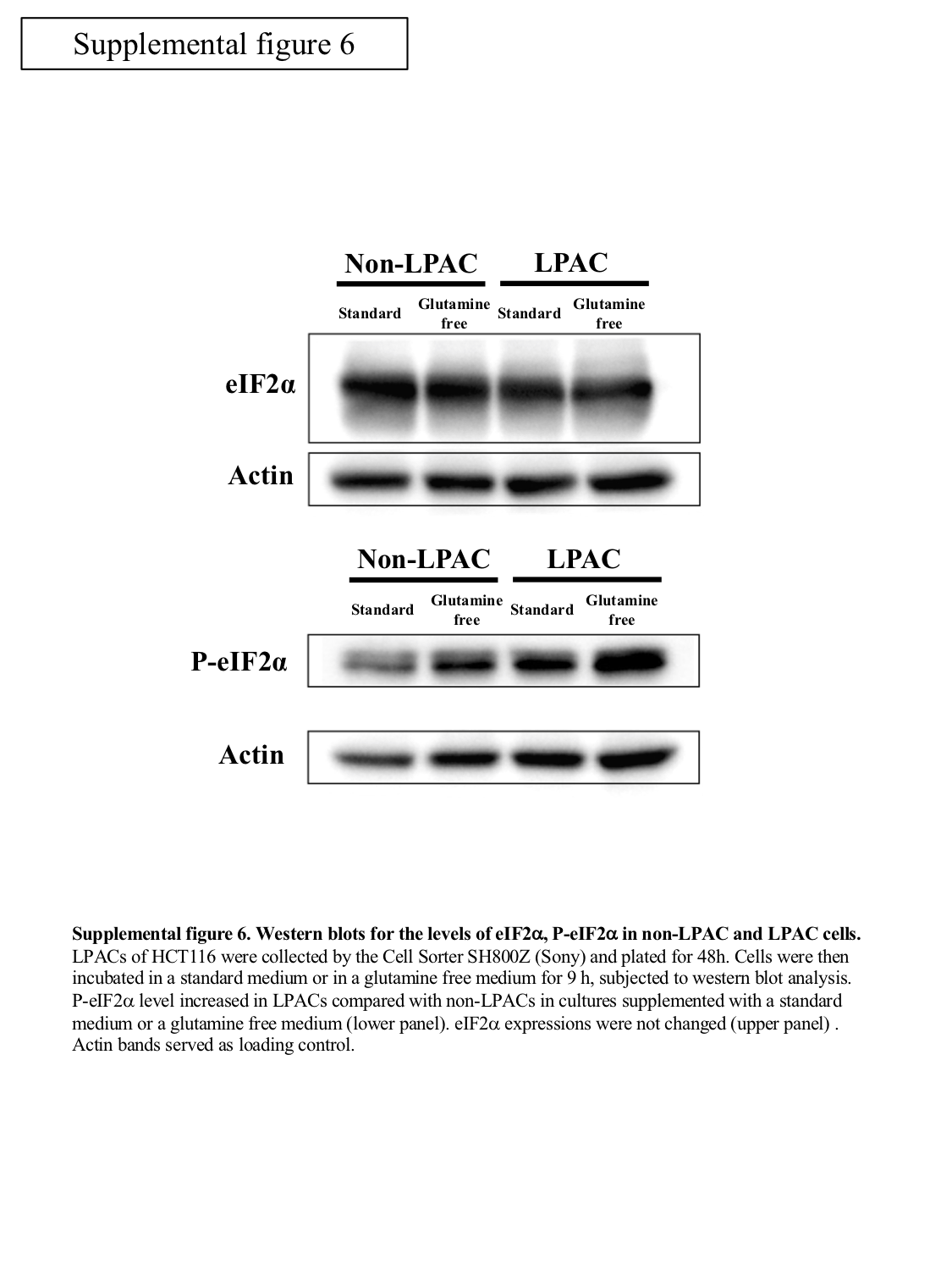

Supplement: Supplementary file 6 — Figure S6 [file AGS3-7-458-s006.tiff]

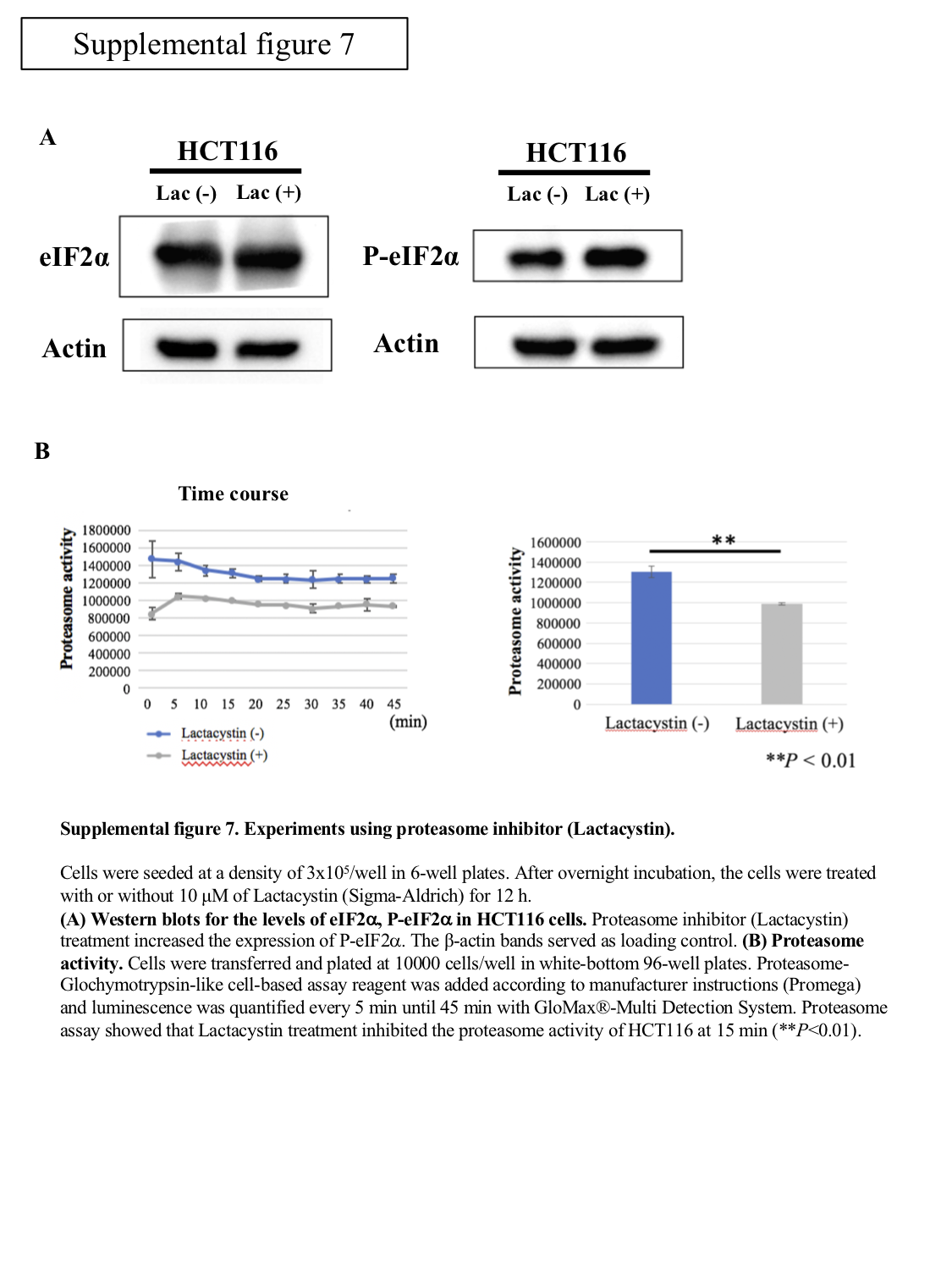

Supplement: Supplementary file 7 — Figure S7 [file AGS3-7-458-s007.tiff]

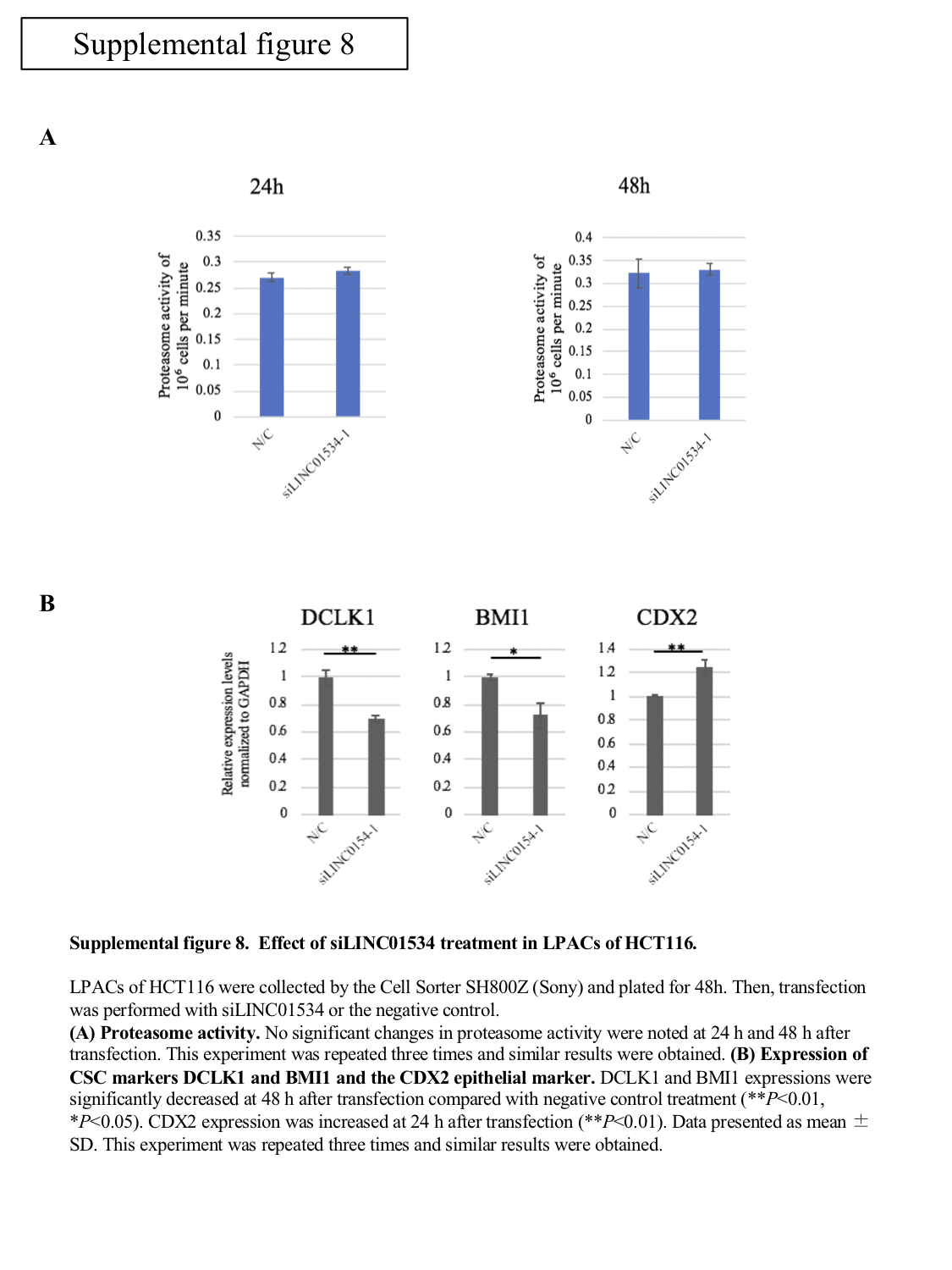

Supplement: Supplementary file 8 — Figure S8 [file AGS3-7-458-s002.tiff]

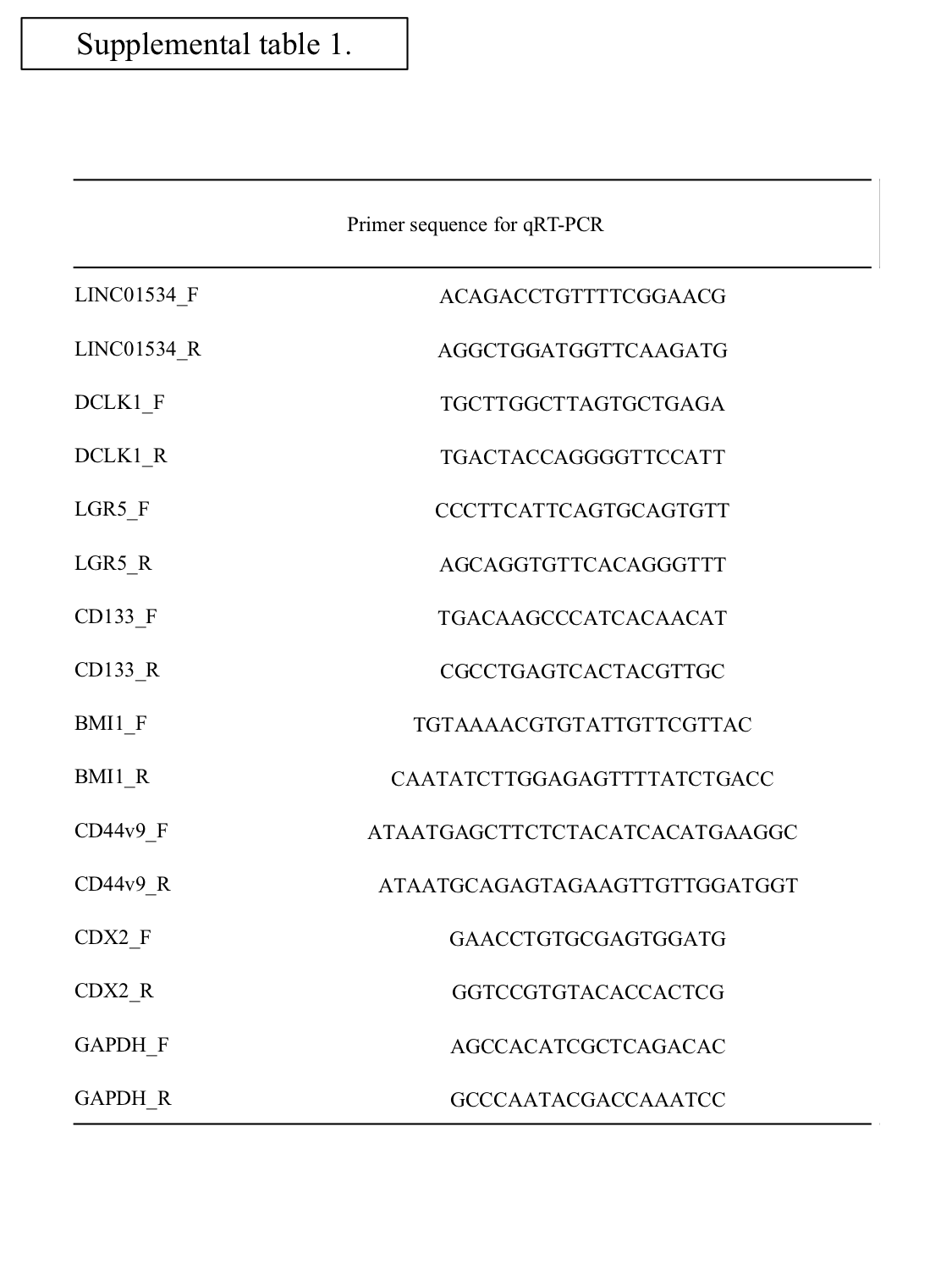

Supplement: Supplementary file 9 — Table S1 [file AGS3-7-458-s001.tiff]

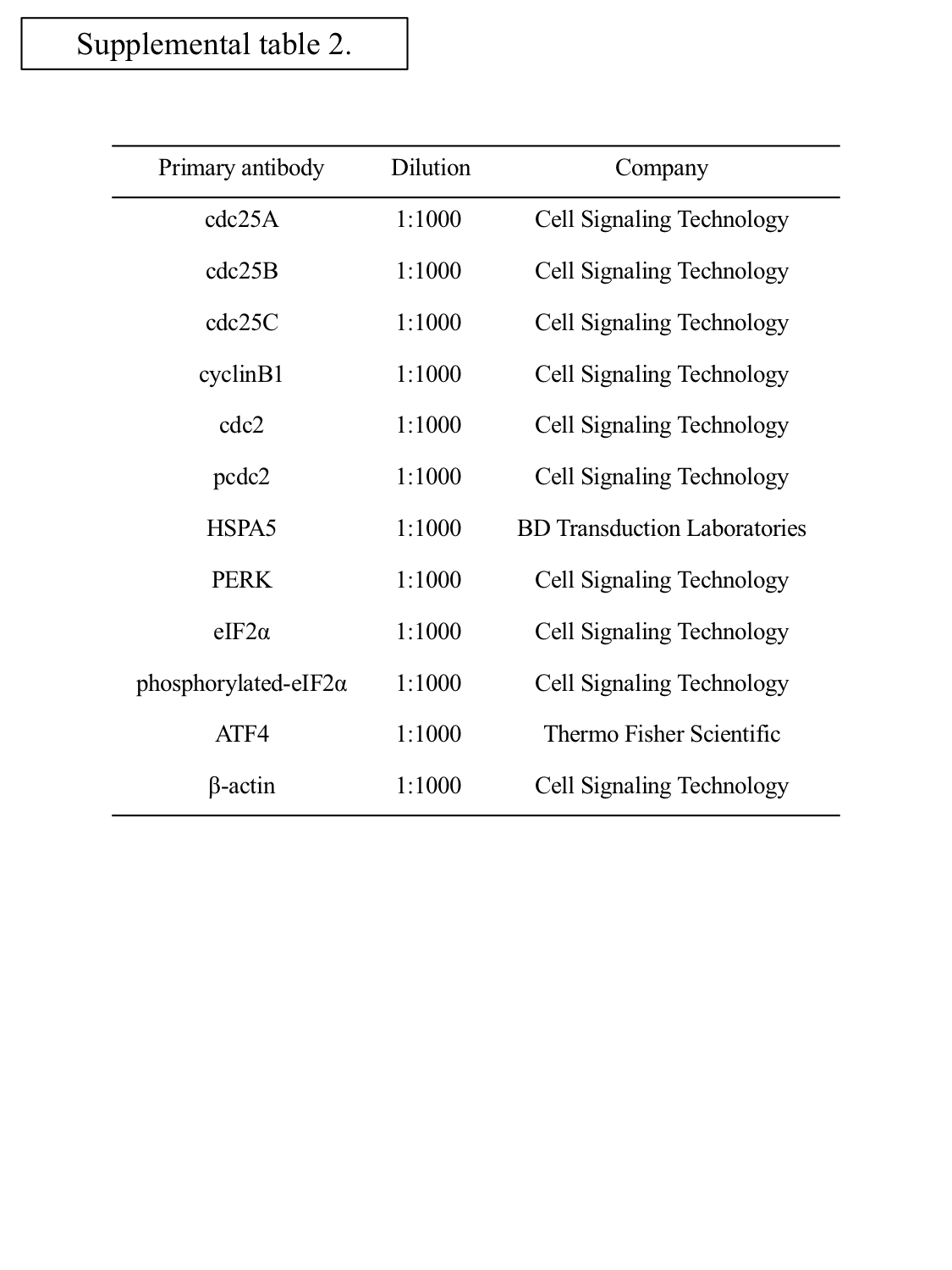

Supplement: Supplementary file 10 — Table S2 [file AGS3-7-458-s010.tiff]
